# Hexokinase II inhibition by 3-bromopyruvate sensitizes myeloid leukemic cells K-562 to anti-leukemic drug, daunorubicin

**DOI:** 10.1042/BSR20190880

**Published:** 2019-09-24

**Authors:** Yogesh Rai, Priyanshu Yadav, Neeraj Kumari, Namita Kalra, Anant Narayan Bhatt

**Affiliations:** Institute of Nuclear Medicine and Allied Sciences, Delhi 110054, India

**Keywords:** 3-Bromopyruvate, cell cycle, Daunorubicin, Hexokinase-II, Leukemia

## Abstract

An increased metabolic flux towards Warburg phenotype promotes survival, proliferation and causes therapeutic resistance, in leukemic cells. Hexokinase-II (HK-II) is expressed predominantly in cancer cells, which promotes Warburg metabolic phenotype and protects the cancer cells from drug-induced apoptosis. The HK-II inhibitor 3- Bromopyruvate (3-BP) dissociates HK-II from mitochondrial complex, which leads to enhanced sensitization of leukemic cells to anti-leukemic drugs. In the present study, we analyzed the Warburg characteristics viz. HK-II expression, glucose uptake, endogenous reactive oxygen species (ROS) level of leukemic cell lines K-562 and THP-1 and then investigated if 3-BP can sensitize the leukemic cells K-562 to anti-leukemic drug Daunorubicin (DNR). We found that both K-562 and THP-1 cells have multi-fold high levels of HK-II, glucose uptake and endogenous ROS with respect to normal PBMCs. The combined treatment (CT) of 3-BP and DNR showed synergistic effect on the growth inhibition (GI) of K-562 and THP-1 cells. This growth inhibitory effect was attributed to 3-BP induced S-phase block and DNR induced G_2_/M block, resulted in reduced proliferation due to CT. Further, CT resulted in low HK-II level in mitochondrial fraction, high intracellular calcium and elevated apoptosis as compared with individual treatment of DNR and 3-BP. Moreover, CT caused enhanced DNA damage and hyperpolarized mitochondria, leading to cell death. Taken together, these results suggest that 3-BP synergises the anticancer effects of DNR in the chronic myeloid leukemic cell K-562, and may act as an effective adjuvant to anti-leukemic chemotherapy.

## Introduction

Glycolytic shift of energy metabolism is one of the fundamental changes that occur in cancer cells. Otto Warburg first described that even in the presence of adequate amount of oxygen, cancer cells increased the glycolytic flux for faster rate of adenosine triphosphate (ATP) production, known as Warburg effect [1]. Likewise, besides the association with distinct genetic lesion’s preferential shift toward glycolytic metabolism is considered as the conserved hallmark in multiple hematological malignancies including acute lymphocytic leukemia (ALL), acute myeloid leukemia (AML), chronic lymphocytic leukemia (CLL) and chronic myeloid leukemia (CML) [2–5]. The M2 phenotype of regulated glycolytic enzyme pyruvate kinase (PKM2) and lactate dehydrogenase A (LDHA), another downstream component of an aerobic glycolytic pathway were found to be over-expressed and have shown a profound effect on acute myeloid leukemia (AML) and CML [5]. Deletion of PKM2 or LDHA manifested by delay in the initiation of leukemogenesis in both BCR-ABL-transformed stem cells (CML) and MLL-AF9-transformed progenitor cells (AML) [6]. Moreover, dysregulation of metabolic pathways through oncogenic transformation like BCR-ABL is one of the important genetic alteration results in reduced oxidative phosphorylation and enhanced glycolysis by expressing the high-affinity GLUT-1 glucose transporter for increased glucose uptake in malignant cells [7–9]. Furthermore, reliance on aerobic glycolysis also observed in leukemia subtypes (B-ALL, T-ALL and AML) [9]. Imatinib is a popular and effective chemotherapeutic drug for leukemia and it has been reported that Imatinib-resistant chronic myelogenous leukemia cells (CML) showed an increased dependency on glycolytic phenotype as compared with Imatinib-sensitive cells [10,11]. Moreover, inhibition of glycolysis by glycolytic inhibitors, such as 3-Bromopyruvate (3-BP), 2-deoxy-d-glucose (2-DG), lonidamine (LND) or the down-regulation of glyceraldehyde-3-phosphate dehydrogenase (GAPDH) through RNA interference leads to sensitization of prednisolone-resistant ALL cell lines to glucocorticoids [12]. As leukemic cells with an increase in glycolytic rate found to display glucocorticoid resistance, and first generation glycolytic inhibitor, 2-DG, enhanced the chemotherapeutic effects of glucocorticoids against leukemic cells with mitochondrial defects. This phenomenon also observed from primary leukemic cells of pediatric leukemia patients [13]. Futhermore, 3-BP treatment potentiated growth arrest and cell death in acute leukemia subtypes, including precursor-B-cell ALL, T-cell ALL and AML [14]. Glycolysis-induced resistance to chemotherapeutic Imatinib in CML is attributed to highly active M2 isoform of pyruvate kinase (PKM2) [6]. These studies support the hypothesis of an increased uptake and assimilation of glucose through glycolyis in leukemic cells. However, so far, studies on the altered glycolytic metabolism of hematological malignancies have been mainly focused on AML and ALL. It has been demonstrated that multidrug-resistant (MDR) leukemic cells exhibit enhanced glycolytic activity and the inhibition of glycolysis potently reverses the resistance of MDR cells to anticancer agents [15]. The increased glycolytic activity has been identified as one of the specific metabolic markers for therapeutic resistance in CML, which often develop protective mechanisms to survive genotoxic treatments [16]. Enhanced glycolysis causes therapeutic resistance by producing ATP at a very rapid rate to repair therapy-induced DNA and other macromolecular damages [17]. Moreover, pyruvate and some other intermediates of glycolysis serve as antioxidant and neutralize therapeutics induced reactive oxygen species (ROS) [18].

Imatinib mesylate and daunorubicin (DNR) are the current frontline treatments for leukemia [19,20]. Imatinib targets ATP binding site of the BCR-ABL protein through competitive inhibition results in apoptosis of the hematopoietic cells [21]. Whereas DNR is known to induce cytotoxic effect through the inhibition of topoisomerase II; furthermore, its ability to intercalate between DNA base pairs results in the consequent blockade of replication and transcription [22]. Because therapeutic resistance in hematological malignancies are attributed in part to up-regulation of glycolysis, therefore recent focus in leukemia treatment research is about adding a glycolytic inhibitor to chemotherapeutics for gaining a promising chemo-sensitization effect.

Hexokinase-II (HK-II), a key enzyme of glycolysis, catalyzes the first essential step of glucose metabolism and the up-regulation of HK-II and its association with mitochondrial outer membrane increases the rate of glycolysis in cancer cells [23]. Apart from that voltage-dependent anion channel (VDAC)-bound HK-II on outer mitochondrial membrane suppresses the formation of mitochondrial permeability transition pores (MPTPs) contributing to the inhibition of apoptosis [23,24]. Therefore, HK-II overexpression not only helps the cancer cells metabolically by increasing the glycolytic rate but also inhibits the apoptosis at mitochondria, making the cells resistant to anticancer drugs [25,26]. It has been found that targeted HK-II inhibition by 3-BP induces apoptosis in cancer cells [23,26]. The increased expression of HK-II in leukemic cells has been demonstrated [27,28], however, the role of HK-II in MDR leukemic cells is still poorly understood. Therefore, we aimed to study the role of HK-II in therapeutic resistance against DNR in leukemic cells K-562. In the present study, we investigated whether HK-II inhibition along with DNR treatment might enhance the sensitizing effect in leukemic cells and ultimately have therapeutic implications for leukemia.

## Materials and methods

### Materials

RPMI 1640, Dulbecco’s Minimum Essential Medium (DMEM) high glucose, Penicillin G, streptomycin, nystatin, Dichlorodihydrofluorescein diacetate (H2-DCFDA), ethylenediaminetetra acetate (EDTA), potassium chloride (KCl), magnesium chloride (MgCl_2_), Protease inhibitor cocktail, HEPES, DAPI, propidium iodide (PI), RNAase, CFSE and 3-BP were purchased from Sigma Chemicals Co. (St. Louis, U.S.A.). DNR was obtained from Neon Laboratories, Ltd., India, whereas Fluo-3-AM, DiOC6, JC-1 and Annexin (Alexa Fluor)/PI procured from Molecular Probes (Eugene, U.S.A.). Lactate and ATP determination kits were obtained from Dialab (Austria) and Thermo Scientific (U.S.A.), respectively. Primary antibodies (VDAC, β-Actin, Bcl-2) were purchased from CST (Danvers, MA, U.S.A.), whereas HK-II, apoptosis inducing factor (AIF) and HRP–conjugated secondary antibodies were procured from Santa Cruz, U.S.A.

### Sources of cell lines

The human cell lines K-562 (CML), THP-1 (acute myeloid leukemia), HEK-293 (human embryonic kidney) were obtained from the NCCS, Pune, India. Whereas, peripheral blood mononuclear cells (PBMCs) were procured from HiMedia (Mumbai, India). K-562, THP-1 and PBMCs were cultured in RPMI-1640 medium and HEK-293 cells in DMEM high glucose, supplemented with 10% FBS, 2 mM glutamine, 1% penicillin and streptomycin, and kept at 37°C in a humidified incubator with 5% CO_2_. Stock cultures were maintained in the exponential growth phase by passaging with their respective growth medium after 2–3 days in T-25 culture flasks (BD Falcon, U.S.A.).

### Drug and inhibitor treatment

Experiments were carried out in 60- and 35-mm tissue culture dishes. Exponentially growing cells were treated with anti-leukemic drug DNR and HK-II inhibitor 3-BP (concentrations are mentioned in the respective figure legends) for 4 h individually and in combination at 37°C in a humidified incubator with 5% CO_2_. After 4-h exposure treatments, medium was removed and supplemented with respective fresh medium and kept in CO_2_ incubator for the further experimental analysis.

### Measurement of glucose uptake, ROS and intracellular calcium

Glucose-uptake endogenous ROS and intracellular calcium were quantified using fluorescent glucose analog 2-NBDG (100 μM; 20 min; 37°C), H2-DCFDA (20 μM; 40 min; 37°C) and Fluo-3AM (5 μM; 30 min; 37°C) respectively; prepared in probe buffer containing 1 mM MgCl_2_ and CaCl_2_ (not added in Fluo-3AM probe buffer) each in phosphate-buffered saline (PBS). After incubation, cells were washed and resuspended in PBS before analysis. The signals were recorded using flow cytometer (BD, FACS Aria™III Cell Sorter, U.S.A.).

### Measurement of HK-II activity

Hexokinase (HK-II) enzymatic activity was measured as previously described [17,29] with some modification. Briefly, K-562 and THP-1 cells were lysed using the buffer containing 50 mM Tris/HCl (pH-8.0), NaCl (150 mM), MgCl_2_ (1.5 mM), sucrose (250 mM), along with protease inhibitor. Two sample aliquots were prepared from each cell line. One aliquot for total and second heated at 45°C for 1 h received only heat-stable HK-I activity. The cell lysates were incubated on ice (30 min), followed by centrifugation (14000 rpm/4°C/10 min). The protein estimation was carried out by BCA method. A total of 20 μg protein from freshly lysed cell supernatant was added to 1 ml of reaction buffer containing 100 mM Tris/HCl, pH 8.0, ATP (10 mM), EDTA (0.5 mM), glucose (2 mM), MgCl_2_ (10 mM), NADP (0.1 mM) and 0.1 U/ml of G6PD (Sigma). Further HK activity determined following the G6P-dependent conversion of NADP into NADPH (OD: 340 nm). HK-II activity obtained after the subtraction (OD values) of HK-I activity from the total HK activity.

### Immunoblotting

Cells were cultured in T-75 flask and treatments were given (as mentioned in figure). Cells were harvested at 4 h after the treatment and lysed in ice-cold RIPA lysis buffer (Tris/HCl: 50 mM, pH 7.4, NaCl (150 mM), NP-40: 1%; Na_3_VO_4_ (1 mM), EDTA (1 mM), PMSF (2 mM), protease inhibitor cocktail, NaF (1 mM)) containing protease inhibitors. Mitochondrial fraction was isolated by conventional differential centrifugation method as described previously [23]. Briefly, K-562 cells were collected at 4 h and washed with cold PBS and re-suspended in a buffer containing 10 mM Tris/HCl, pH 7.5, MgCl_2_ (1.5 mM), NaCl (10 mM), EDTA (1 mM), sucrose (70 mM), mannitol (210 mM) and protease inhibitors. Samples were incubated on ice and homogenized with 15 strokes in a 2-ml glass homogenizer. Further samples were centrifuged twice (1500×***g***; 4°C; 5 min) to remove nuclei and cell debris followed by supernatants centrifugation (1500×***g***; 4°C; 5 min) to separate the mitochondrial fraction and cytosolic fraction. Immunoblotting of HK-II and VDAC as loading control carried out in mitochondrial-enriched fraction whereas HK-II, BCL-2, AIF performed in whole cell lysate. The protein concentration in samples was determined by BCA protein assay kit. Proteins (50 μg) were resolved on 10–15% SDS/PAGE (depending on the molecular weight of the proteins) and electroblotted on to PVDF membrane (MDI). The membrane was then incubated in 5% BSA (according to manufacturer’s protocol) for 1 h followed by primary antibody incubation in the dilution of (1:1000). Membrane was washed with 1× TBST (Tris-buffered saline) followed by incubation with the appropriate HRP–conjugated secondary antibody dilution (1:5000) for 2 h. After TBST washing, further blots were developed using ECL chemiluminescence detection reagent (Biological Industries, Israel).

### Cell number estimation and cell cycle analysis

The cells were plated in 60-mm culture dishes (0.15 × 10^6^ cells/4 ml/PD) kept at 37°C in a humidified incubator with 5% CO_2_. Next day, drug and inhibitor treatment (4-h exposure) was given; further at desired time points cells were terminated and counted with a Neubauer-improved counting chamber (Paul Marienfeld GmbH & Co. KG, Germany) under 10× objective, and 10× eyepiece magnification with compound light microscope (Olympus CH30, Japan). For the cell cycle analysis at respective time points (4 and 24 h) cells were harvested and fixed in 70% ethanol and stored at −20°C (overnight). Further, cells were washed and re-suspended in PBS containing RNase A (200 μg/ml; 30 min; 37°C) and stained with PI (200 μl of PI 50 μg/ml; 15 min; 37°C). Samples acquisition and data analysis were carried out using flow cytometry (BD, FACS Aria™III Cell Sorter, U.S.A.).

### Estimation of Synergistic Index

Cell growth inhibition (GI) data were obtained after normalization of individual and combined treatment (CT) groups with respective control. A Synergistic Index (SI) was then calculated as previously described [30] with the GI% (at 48 h) ratio of drug and inhibitor CT and treatment alone (TA) (SI = GI% CT/GI% TA). SI ≥ 1.00 indicates synergistic response, i.e. GI of CT was greater than TA.

### Estimation of lactate, ATP and measurement of mitochondrial complex-II activity

Glycolytic and mitochondria-associated energy metabolism was examined in terms of lactate and ATP production respectively. Lactate and ATP were quantified using Lactate enzymatic, UV kit (Dialab, Austria) and ATP bioluminescence assay kit (Thermo Scientific, U.S.A.) as per the manufacturer’s protocol at 4 and 24 h following treatment. Mitochondrial complex-II activity was measured at 4 and 24 h following treatment in K-562 cells. Succinate dehydrogenase (SDH)-dependent reduction of MTT into formazan was examined in the mitochondrial fraction as described previously [31]. Formazan was quantified using formazan standard and presented as differential fold change among treatment groups with respective control.

### CFSE proliferation assay and apoptosis detection

Cell proliferation assay was performed using CFSE probe (Sigma) according to manufacturer’s protocol. Briefly,cells were incubated with 5 μM CFSE in medium supplemented with 2% serum at room temperature for 20 min (with continuous rolling). Further cells were washed with respective growth medium (without probe) and seeded at a density of 0.25 × 10^6^ and kept at 37°C in CO_2_ incubator. After 24-h treatments were given, further cells were terminated at 4 and 24 h post treatment, followed by measurement using flow cytometry. For apoptosis detection, cells were terminated at 24 h (after the treatment) and quantified by Annexin V/PI kit (Invitrogen) according to manufacturer’s protocol using flow cytometry.

### Nuclear fragmentation and 53BP-1 foci formation assay

Nuclear fragmentation assay was performed as described earlier [32]. Briefly, cells were seeded in 96-well plate at a density of (0.01 × 10^6^ cell/200 μl/well) and kept at 37°C in CO_2_ incubator. Next day treatment was given (as described above) and after 24 h cells were terminated by centrifugation (100×***g***; 10 min; 37°C). Cells were fixed in 96-well plate with 4% paraformaldehyde for 10 min followed by washing with PBS and permeabilization with 0.1% Triton X-100 (10 min). Further cells were washed with PBS and stained with DAPI (4,6-di-amidino-2-phenylindole) for 5 min. Images were captured using fluorescence microscope (Olympus IX 51 fluorescence microscope, Japan). 53 P-1 foci formation was performed in HEK transfected cells. The 53 P-1-GFP plasmid was a kind gift from Dr. Deepak Saini’s Laboratory, Indian Institute of Science (IISc), Bangalore, India. A permanently transfected cell line was established by transfecting this plasmid. For imaging, cells were seeded in 35-mm dishes containing coverslips at a density of (0.075 × 10^6^ cells/2 ml/PD). Next day, treatment was given and cells were terminated at 4 and 24 h post treatment and images (GFP fluorescence) were captured to observe the foci formation using fluorescence microscope (Olympus IX 51 fluorescence microscope, Japan) under 10× (objective) × 10× (eyepiece) magnification.

### Determination of mitochondrial membrane potential using DiOC6 and JC-1

Measurement of treatment-induced alteration in mitochondrial membrane potential determined by DiOC6 staining. At 4 and 24 h post treatment cell were terminated and stained with DiOC6 (100 nM; 30 min; 37°C), followed by PBS washing and resuspended in PBS before analysis. Fluorescence signal were recorded using flow cytometer. Simultaneously microscopic analysis was carried out by membrane potential sensitive probe JC-1. K-562 suspension cells were seeded with the density of (0.01 × 10^6^ cells/well). At respective time points (4 and 24 h) following treatment cells were pellet down in 96-well plate and stained with JC-1 (10 mg/ml; 30 min; 37°C). Further cells were washed with cold PBS followed by centrifugation (100×***g***; 10 min; 37°C). Fluorescence image were captured in 96-well plate using fluorescence microscope (Olympus IX 51 fluorescence microscope, Japan) under 10× (objective) × 10× (eyepiece) magnification.

### Statistical analysis

Student’s *t* test was performed for all the statistical analysis of experiments. Values are presented as the means ± standard deviation (SD) obtained from triplicates or quadruplicates experiments (mentioned in respective figure legends) and significance was set as *P*-value less than 0.05 between the groups.

## Results

### Myeloid leukemic cells exhibit high glycolytic and HK-II activity

**Figure 1:**
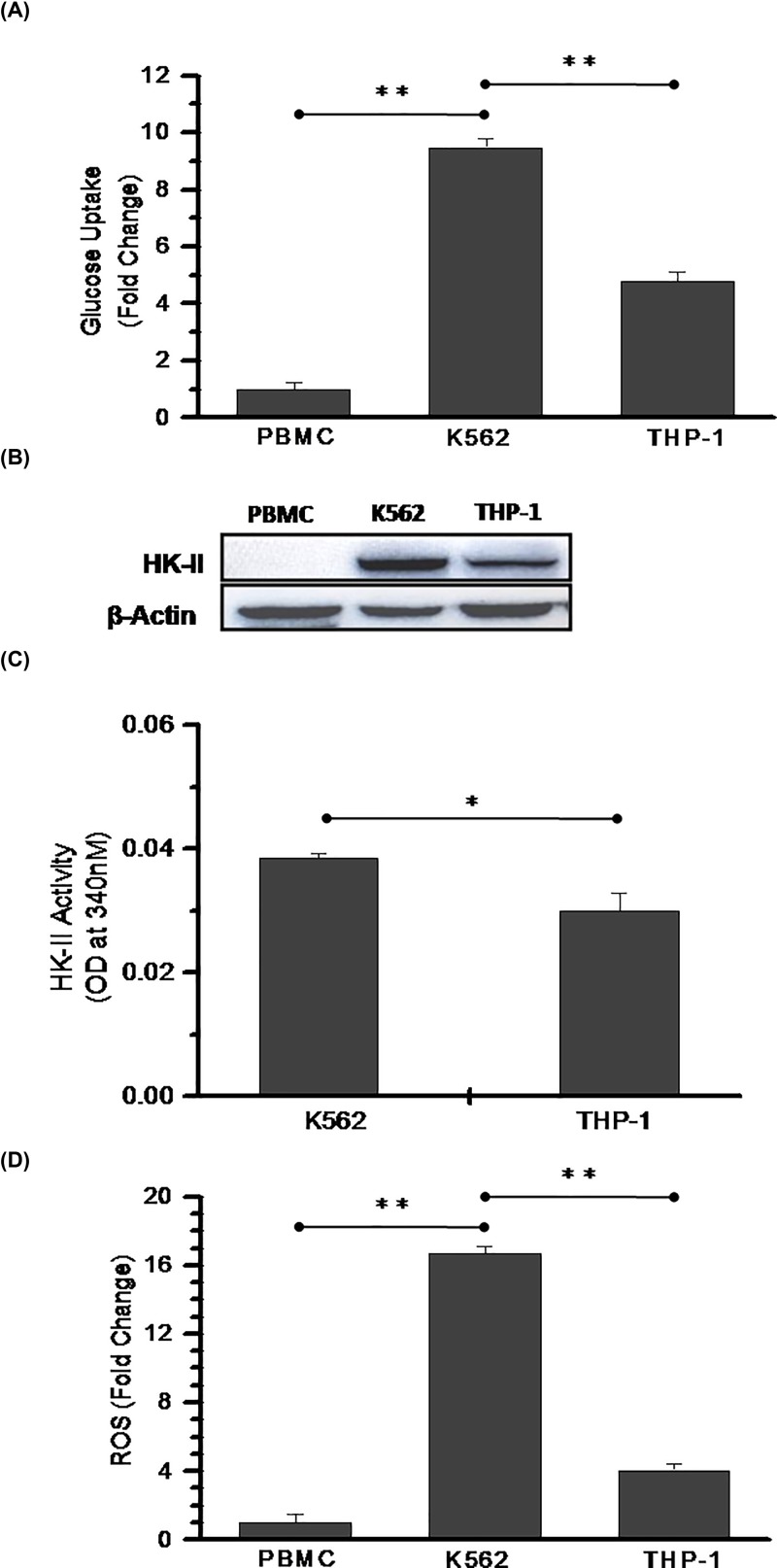
Increased HK-II activity promoted glycolytic phenotype in leukemic cells (**A**) Glucose uptake assay was performed using 2-NBDG in normal PBMCs and leukemic K-562 and THP-1 cells. Bar diagram represents fold change with respect to PBMCs. (**B**) Immunoblotting analysis for HK-II expression in the normal PBMCs, K-562 and THP-1 cells. (**C**) HK-II enzyme kinetics was performed in K-562 and THP-1 cells. Data presented here are NADP to NADPH conversion at 15 min (OD at 340 nm). (**D**) The endogenous ROS measured using H2-DCFDA and graph presented as relative fold change of PBMCs. Data are presented as mean ± SD (*n*=3) and statistical significance (*P<0.05; **P<0.01).

We performed glucose uptake assay to observe the glycolytic dependency of K-562 (CML), THP-1 (Acute myeloid leukemia) cell lines and compared with the normal PBMCs (peripheral blood mononuclear cells). Both leukemic K-562 and THP-1 cells showed significantly high glucose uptake of 9- and 5-fold respectively as compared with normal PBMCs (Figure 1A). High glucose uptake of cancer cells is supported by enhanced levels of HK-II [33] therefore, we checked HK-II protein level in these cells. We could not detect HK-II expression in PBMCs whereas, prominent expression was observed in K-562 and THP-1 cells (Figure 1B). In order to validate the levels of HK-II expression, we performed HK-II enzyme activity assay in K-562 and THP-1 cells. We noted significant increase in HK-II activity of K-562 cells as compared with THP-1 cells (Figure 1C). The increased endogenous ROS in cancer cells causes an increase in glycolytic activity and pathological states of both AML and CML are also accompanied by excessive ROS production [34,35]. Therefore, next we measured endogenous ROS level to find if an increased glucose metabolism correlated with deregulated redox homeostasis of these leukemic cells. Both K-562 and THP-1 cells showed 17- and 4-fold elevated ROS production respectively as compared with normal PBMCs (Figure 1D). In K-562 cells multifold high endogenous ROS level (Figure 1D) correlated with increased glycolytic phenotype in K-562 cells (Figure 1A). These results suggests that chronic myeloid leukemic cells, i.e. K-562 exhibit an increased glycolysis as compared with the acute myeloid leukemic cells (THP-1).

### HK-II inhibition using 3-BP in combination with DNR showed synergistic GI response in myeloid cells

**Figure 2:**
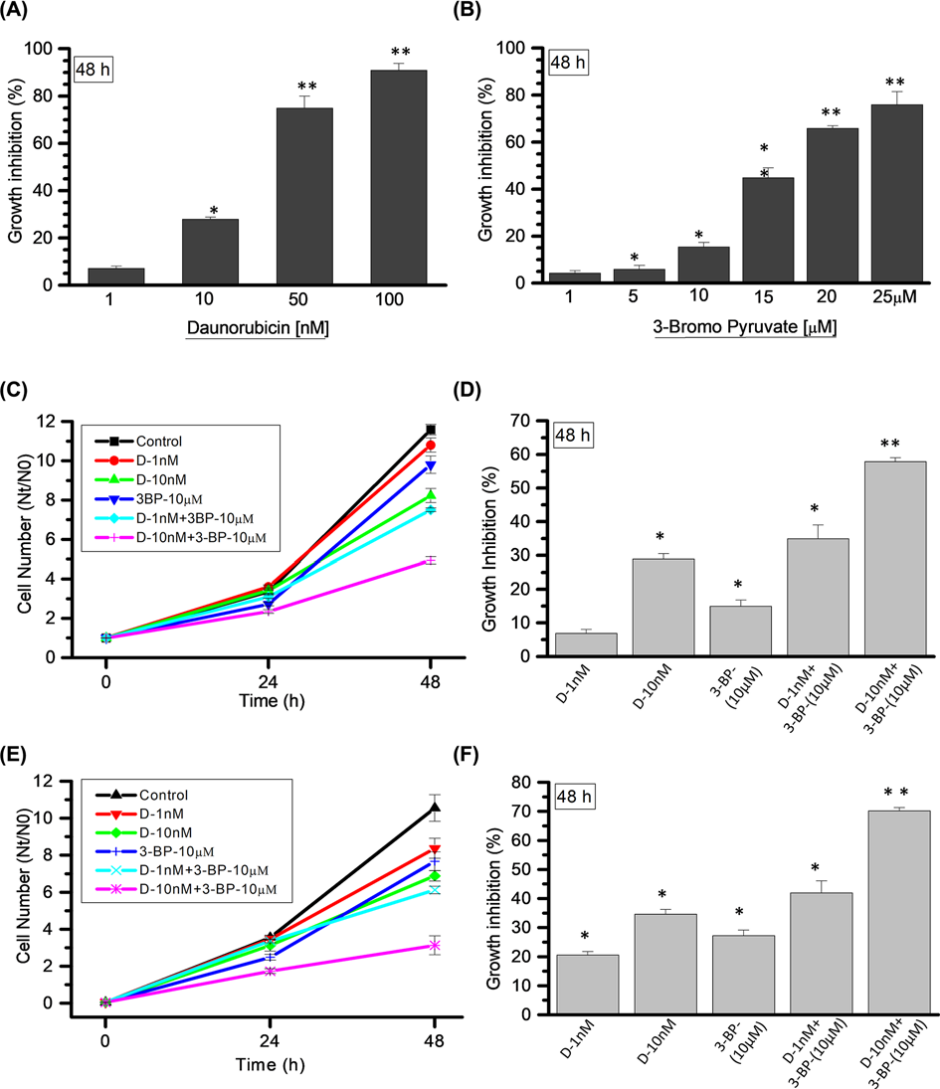
Combined treatment leads to synergistic effect on GI in myeloid cells (**A**) DNR and (**B**) 3-BP; post treatment cell numbers estimated at 48 h for the screening of different DNR and 3-BP concentrations (as mentioned in graph). Graph presented here as GI (%) compared with control in K-562 cells. (**C**) Cell number estimated using combination and TA as mentioned in graph at indicated time points in K-562 cells. (**D**) Treatment-induced GI quantified and presented at 48 h (derived from (C)). (**E,F**) Cell number and GI estimated and presented in THP-1 cells under similar experimental conditions used in K-562 cells. Data are presented as mean ± SD (*n*=4) and statistical significance (*P<0.05; **P<0.01).

Previous results revealed that K-562 cells utilize more glucose and acquire an increased HK-II-mediated glycolytic phenotype. Therefore, we investigated, whether inhibition of glycolysis using HK-II inhibitor 3-BP can enhance the cytotoxic effect of anticancer agent (DNR) in K-562 cells. We screened the cytotoxic concentration range of DNR (1–100 nM) and 3-BP (1–25 μM) by cell growth assay (Figure 2A,B) to identify the optimum concentration with less cytotoxic effect in K-562 cells. At 48 h (hours), the DNR concentration of 1 and 10 nM showed 7 and 29% cell GI, respectively (Figure 2A), whereas, 10 μM of 3-BP was selected with 15% GI (Figure 2B). Further these selected concentrations of DNR and 3-BP were examined alone and in combination to observe the effect of HK-II inhibition on growth inhibitory effects of DNR in K-562 cells. Growth kinetics assay was performed at 0, 24 and 48 h and GI was quantified followed by comparative analysis at 48 h among the treatment groups (Figure 2C). The DNR at (1 nM) and 3-BP (10 μM) showed 7 and 15% cell GI, respectively, whereas, CT (DNR-1nM + 3-BP-10 μM) resulted in 35% GI in K-562 cells (Figure 2D). Furthermore, CT of 10 nM DNR with 10 μM 3-BP inhibited 58% cell growth as compared with 29 and 15% in drug and inhibitor alone, respectively (Figure 2D). We also investigated the growth inhibitory effect of DNR and CT in THP-1 cells under similar experiential conditions (Figure 2E). The CT showed 42% (DNR-1 nM + 3-BP-10 μM) and 70% (DNR-10 nM + 3-BP-10 μM) GI as compared with 21% (DNR-1 nM) and 35% (DNR-10 nM), respectively in DNR alone (Figure 2F). The CT induced GI was found to be synergistic in both K-562 and THP-1 cells as shown in Table 1. Since in primary screening, we found HK-II significantly up-regulated in K-562 cells as compared with THP-1 cells, therefore, in order to elucidate the underlying mechanism of HK-II-mediated leukemic cell sensitization, further experiments were carried out in K-562 cells.

**Table 1:** Analysis of SI from CT (DNR+3-BP) *vs* DNR TA in myeloid cells

Sl. no.	Cell lines	Drug treatments	SI calculations for CT	SI value
1	K562	DNR-1 nM	SI = GI% (DNR-1 nM+3-BP-10 μM)/GI% (DNR-1 nM) = **35/7**	5 (>1)
		DNR-10 nM	SI = GI% (DNR-10 nM+3-BP-10 μM)/GI% (DNR-10 nM) = **58/29**	2 (>1)
2	THP-1	DNR-1 nM	SI = GI% (DNR-1 nM+3-BP-10 μM)/GI% (DNR-1 nM) = **42.03/20.68**	2.03 (>1)
		DNR-10 nM	SI = GI% (DNR-10 nM+3-BP-10 μM)/GI% (DNR-1 nM) = **70.26/34.72**	2.02 (>1)

### Combined treatment of DNR and 3-BP inhibits glycolysis and ATP production

**Figure 3:**
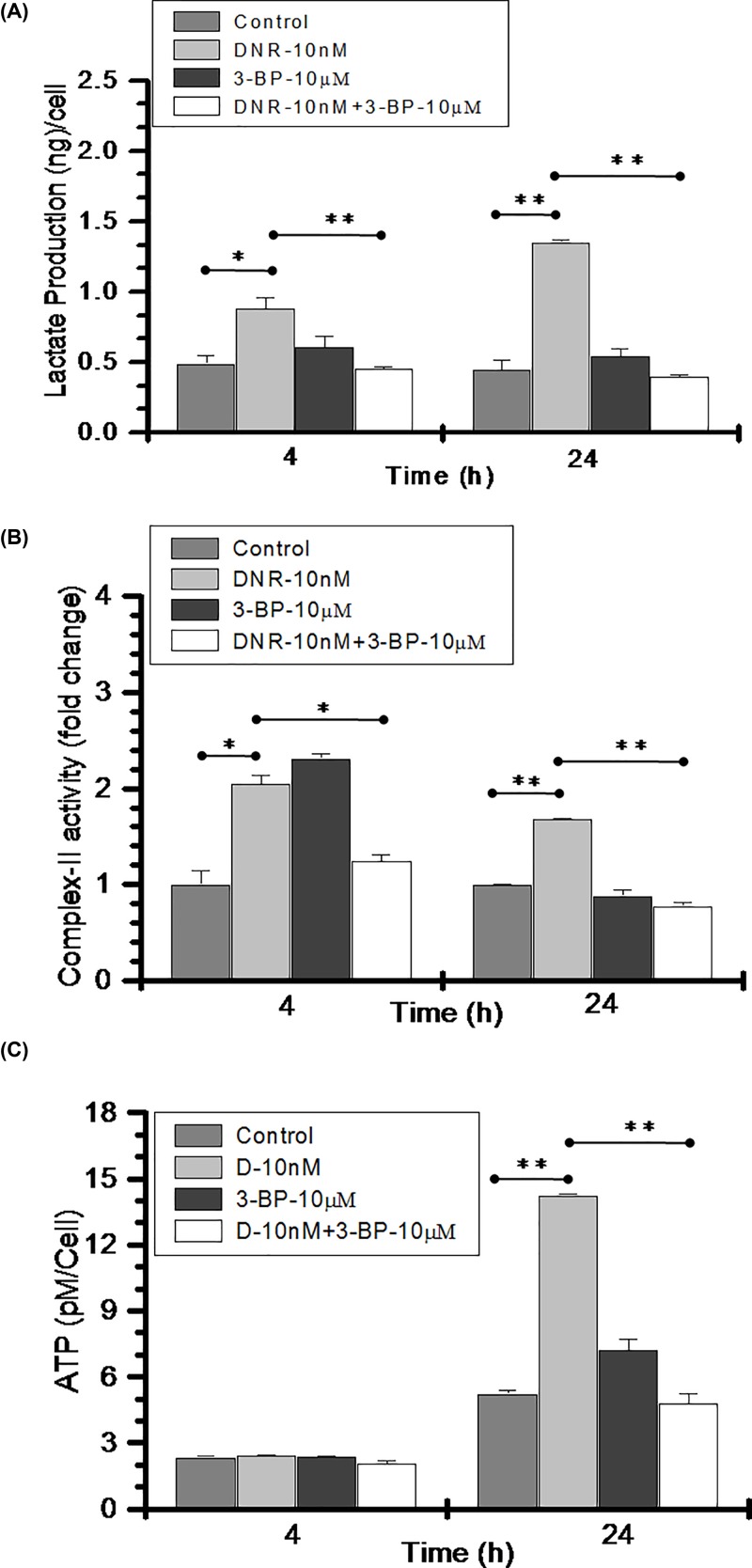
Combined treatment induced inhibition of energy metabolism in K-562 cells (**A**) Lactate production was quantified in control, DNR (10 nM), 3-BP (10 μM) and CT (DNR-10 nM + 3-BP-10 μM) and presented as (ng/cell) at indicated time points. (**B**) Mitochondrial complex-II activity is presented as fold change with respect to control. (**C**) Treatment-induced ATP production was quantified in all the treated groups and presented as pM/cell at indicated time points. Data are presented as mean ± SD (*n*=3) and statistical significance (*P≤0.05; **P≤0.01).

Next, we investigated the status of glycolysis, mitochondrial complex-II activity and ATP level following drug treatments. Lactate production was quantified at 4 and 24 h. DNR treatment (10nM) induced significantly high lactate production at 4 h (0.88 ng/cell) and 24 h (1.35 ng/cell) as compared with control cells 0.48 ng (4 h) and 0.44 ng/cell (24 h) (Figure 3A). On the other hand, CT of DNR (10nM) and 3-BP (10μM) resulted in 0.45ng/cell and 0.39ng/cell at 4 and 24 h respectively, which is significantly low as compared with DNR TA. Further, to analyze the energy metabolism at mitochondrial level, we measured the complex II (succinate dehydrogenase) activity of electron transport chain in drug alone and CT group. Interestingly, at 4 h the complex II activity was significantly high in both DNR (2 fold) and 3-BP (2.3 fold) treatments and comparable with control in the CT (1.23-fold). However, at 24 h significant increase in the complex-II activity remained persistent in the DNR treatment with nearly 1.7-fold with respect to control whereas, substantially low activity was noted in the combined treated cells as compared with control and DNR treatment (Figure 3B). Furthermore, we quantified ATP in these treatment groups. In line with lactate production and mitochondrial energy metabolic activity, we found nearly 3 fold increase in the ATP level of DNR treated cells at 24 h which was significantly reduced in the CT group (Figure 3C). However, no change in the ATP levels were observed in any treatment groups at 4 h. Together these findings suggest that DNR further induces glycolytic metabolism and energy production in highly glycolytic K-562 cells, however CT inhibits the cellular energy metabolism in these cells.

### Combined treatment of DNR and 3-BP induces cell cycle arrest and impede the proliferation rate

**Figure 4:**
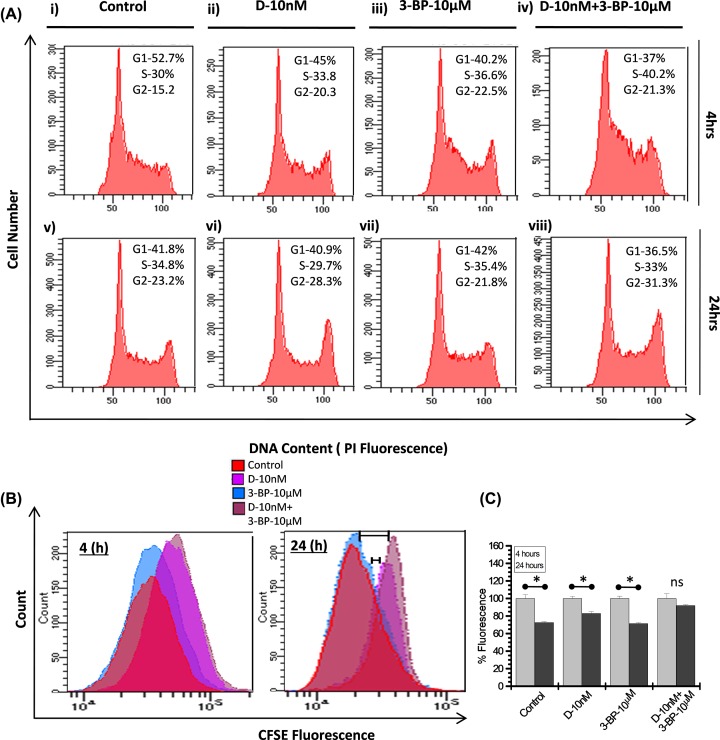
Combined treatment induced cell cycle arrest and inhibition of proliferation (**A**) Flow cytometric analysis of DNA content at indicated time points carried out in K-562 cells treated alone as well as combination of DNR (10 nM) and 3-BP (10 μM). Cell cycle percentage distribution of G_1_, S and G_2_-M populations are indicated. (**B**) Analysis of cell proliferation was carried out by CFSE labeling in K-562 cells. Overlay graph obtained and presented as treatment induced population shift at indicated time points. (**C**) Graph presented as CFSE fluorescence reduction (%) at indicated time points. Data expressed as mean ± SD (*n*=4) and statistical significance (*P<0.05), p = ns (no significance).

We analyzed cell cycle phase distribution to elucidate the reasons in cell growth delay following 3-BP and DNR treatment in K-562 cells. The flow cytometric measurement of DNA content clearly showed the S phase block in 3-BP and CT only (Figure 4A) at 4 h. We observed nearly 22% and 34% increase in S-phase cell population in both 3-BP and CT respectively (Figure 4A(iii,iv)) with respect to control. Further S-phase block was accompanied by G_2_-M phase block in all the treatment groups, at 4 h (Figure 4A(ii–iv)). However, at 24 h after treatment the G_2_-M block was observed predominantly with an increase in 22 and 35% in DNR and CT, respectively (Figure 4A(vi,viii)). This observation was further validated by CFSE proliferation assay. We examined treatment-induced proliferation by labeling cells with CFSE and evaluated by flow cytometry. Fluorescence shift representing proliferation at 4–24 h (post treatment) presented as overlay graph (Figure 4B). The cells showing high fluorescence are actually representing low proliferation. Visibly high fluorescence meaning low proliferation was observed in DNR and CT with respect to control. Although, the proliferation pattern of 3-BP is nearly similar to the control but the CT showed slower proliferation rate than DNR alone at 24 h. We observed varied extent of significant reduction in fluorescent cell population among control and treatment groups, which showed significant fluorescence shift between 4 and 24 h (Figure 4C), except the CT (DNR+3BP), which showed non-significant reduction (*P*=0.126) at 24 h suggesting very minimal cell proliferation as compared with DNR and control cells (Figure 4C). Together these results suggest, HK-II inhibition by 3-BP in combination with DNR leads to the inhibition of cell proliferation marked by initial S-phase block at 4 h and later G_2_-M block at 24 h (Figure 4A(iv,viii)).

### Mitochondrial dissociation of HK-II by 3-BP potentiates the DNR-induced apoptosis in K-562 cells

**Figure 5:**
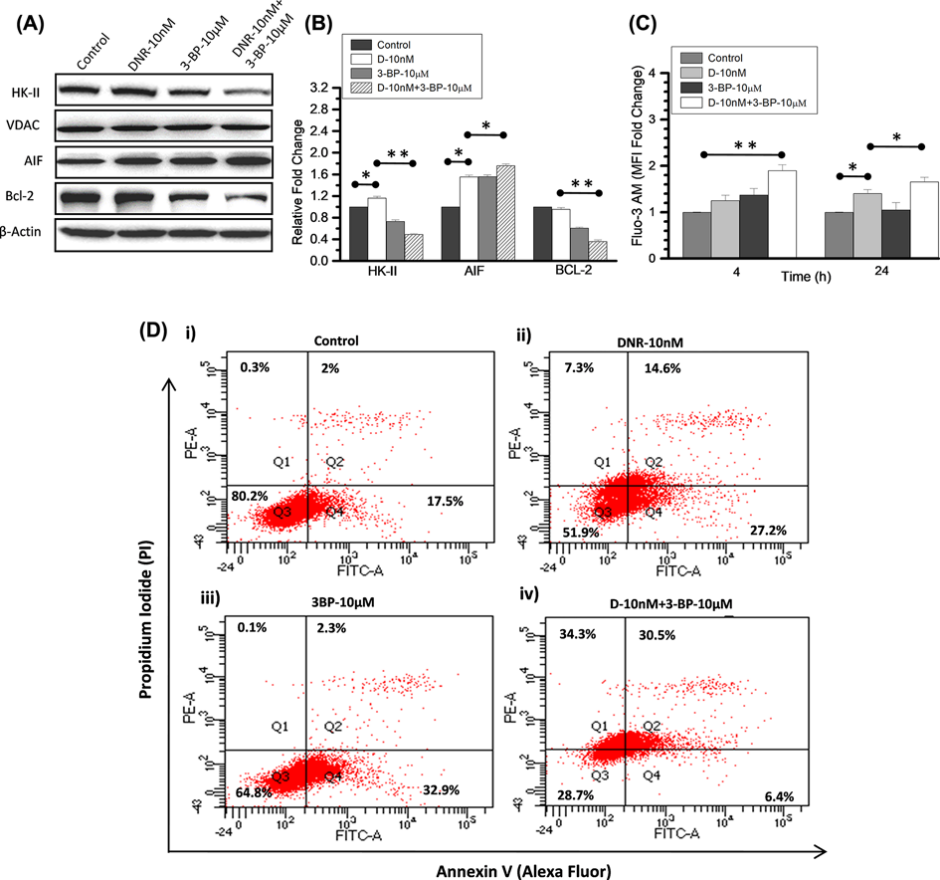
Mitochondrial HK-II dissociation accompanied by increased extracellular calcium and apoptosis in combined treatment (**A**) Immunoblotting was performed (at 4 h) to examine the treatment-induced changes in HK-II expression level and its correlation with initial apoptotic signals in K-562 cells. HK-II protein expression presented here in the mitochondrial enriched fraction of control, DNR (10 nM), 3-BP (10 μM) and their CT compared with loading control VDAC. Apoptosis inducing factor (AIF) and anti-apoptotic protein (Bcl-2) were checked in the whole cell lysate and compared with respective β-actin. (**B**) Immunoblotting densitometry was performed (using ImageJ software) in the indicated proteins and graph presented as relative fold change compared with control. (**C**) Intracellular calcium measurement was carried out using Fluo-3AM at indicated time points after the DNR and 3-BP treatment in combination and alone. Graph presented here as relative fold change in the mean fluorescence intensity (MFI) with respective control. Data are expressed as mean ± SD (*n*=3), statistical significance (*P<0.05; **P<0.01 vs. untreated control cells). (**D**) Flow cytometric analysis using Annexin V (Alexa fluor)/PI in the combined (DNR and 3-BP) and TA in K-562 cells at 24 h showing retention of different apoptotic phase events (%) presented as quadrants (Q1–Q4).

Next, we investigated the correlation between HK-II inhibition and DNR-induced cell growth delay in K-562 cells. We checked whether DNR and 3-BP co-treatment potentiated the cytotoxicity, which resulted in synergistic growth delay with respect to individual treatment and control. We know, the mitochondrial bound HK-II fraction inhibits the drug-induced apoptosis thereby leading to resistance in cancer cells [24,25]. Therefore, we examined the mitochondrial fraction of HK-II in control and treatment groups using immunoblotting. After 4-h treatments, we observed distinct decrease in the mitochondria-associated HK-II levels of CT (DNR+3BP) as compared with control and DNR (Figure 5A). Furthermore, we validated our observation by densitometry (*n*=3) and CT showed nearly 0.5-fold decrease in HK-II level, whereas 1.16-fold increase was observed in DNR as compared with control. However, 3-BP treatment also reduced the mitochondrial HK-II level (Figure 5A) by 0.73-fold as compared with control (Figure 5B). Moreover, prominent decrease in anti-apoptotic protein (BCL-2) and an increased level of AIF was observed in whole cell lysate of co-treated sample (Figure 5A), suggesting enhanced apoptotic signaling. Since mitochondrial HK-II dissociation accompanied by opening of MPTP and cytochrome *c* release followed by increased intracellular calcium [33,36], so, we measured extracellular calcium using calcium binding probe FLUO-3AM. CT showed significant increase in extracellular calcium at both 4 and 24 h (post treatment) as compared with respective control and TA (Figure 5C). Further, we analyzed the apoptosis in K-562 cells at 24 h, using Annexin V/PI assay. The DNR and 3-BP alone treatment (Figure 5D(ii,iii)) showed noticeable increase in early apoptotic cells of 27.2 and 32.9% (Annexin+ve/PI−ve; Q-4), respectively. However in the CT, 31% cells were observed in late apoptotic phase (dual positive quadrant) and 34% cells were necrotic (PI positive) (Figure 5D(iv)). This result was in line with the previous observation of reduced mitochondrial HK-II level, cytosolic Bcl-2, and increased level of cytosolic AIF and calcium in the CT. These findings suggest that 3-BP significantly enhanced the cytotoxic impact of DNR on K-562 cells as compared with DNR alone.

### HK-II inhibition augmented DNA damage efficiency of DNR

**Figure 6:**
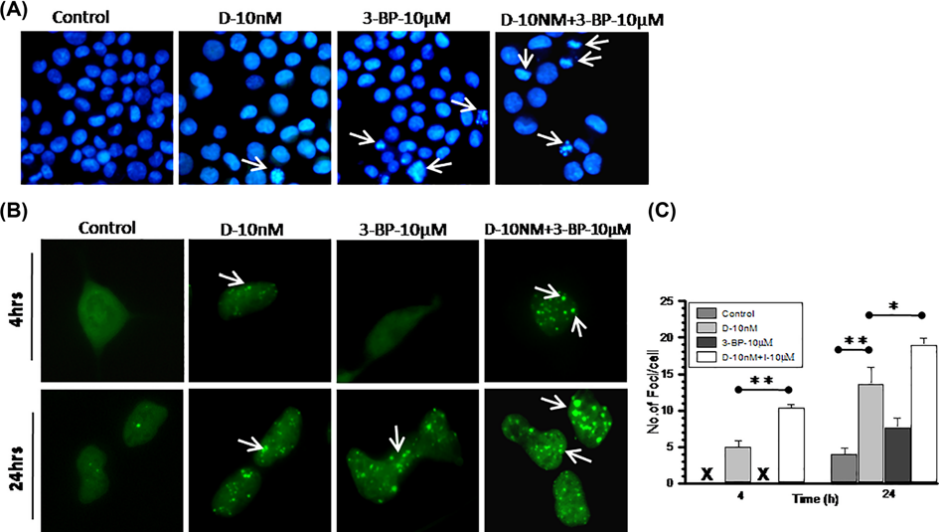
DNR-induced DNA damage significantly enhanced with 3-BP combined treatment (**A**) Nuclear fragmentation assay was performed to visualize the treatment induced alteration in nuclear morphology in K-562 cells. At 24 h, post treatment of DNR and 3-BP alone and in combination cells were fixed and stained with DAPI. Photomicrograph showing variation in nuclear size, condensed chromatin, and fragmented nuclei compared among treatment groups. Images were captured under 10× (objective) × 10× (eyepiece) magnification. (**B**) DNA damage analysis was carried out at indicated time points post-treatment of DNR and 3-BP alone and combination in HEK-293 cells transfected with DNA damage marker 53-BP1-GFP. In photomicrograph, arrow indicates foci formation in damaged cells. (**C**) From image analysis number of foci counted and graph presented as number of foci per cell compared among treatment groups. All the images were captured under 20× (objective) × 10× (eyepiece) magnification. Quantitative data are expressed as mean ± SD (*n*=3) and statistical significance *P<0.05; **P<0.01 vs. untreated control cells.

The DNR induced DNA damage leading to nuclear fragmentation showed linear correlation with the induction of apoptosis in leukemic cells [37,38]. Therefore, we analyzed DNR-induced DNA damage and performed nuclear fragmentation assay to investigate the role of DNA damage in enhanced cytotoxic potency of combined DNR and 3-BP treatment. Photomicrographs showed very few nuclei with fragmented DNA morphology in DNR and 3-BP treated cells compared with the CT (Figure 6A). Further, to validate our results, we carried out 53BP1 foci formation assay in 53BP1 transfected normal human embryonic kidney cells (HEK-293) cells. 53BP1 is a substrate for ataxia telangiectasia mutated (ATM) kinase and considered as classic late DNA damage response (DDR) marker [39]. Photomicrograph showed foci formation linked with treatment-induced DNA damage in HEK-293 cells (Figure 6B). At initial 4 h foci formation was lacking in control and 3-BP treated cells, whereas surprisingly CT showed significant increase in per nuclei foci formation as compared with DNR TA (Figure 6B,C). At 24 h (post treatment) foci formation was visualized in 3-BP treated cells also, whereas number of foci was significantly increased in CT as compared with both 3-BP and DNR TA (Figure 6B,C). Evidently, larger sizes of foci were also clearly viewed at 24 h only in CT (Figure 6B). These findings suggest that HK-II inhibition using 3-BP significantly potentiate the DNR-induced DDR which also manifested by enhanced apoptosis in K-562 cells**.**

### Combined treatment (DNR and 3-BP) induces mitochondrial hyperpolarization

**Figure 7:**
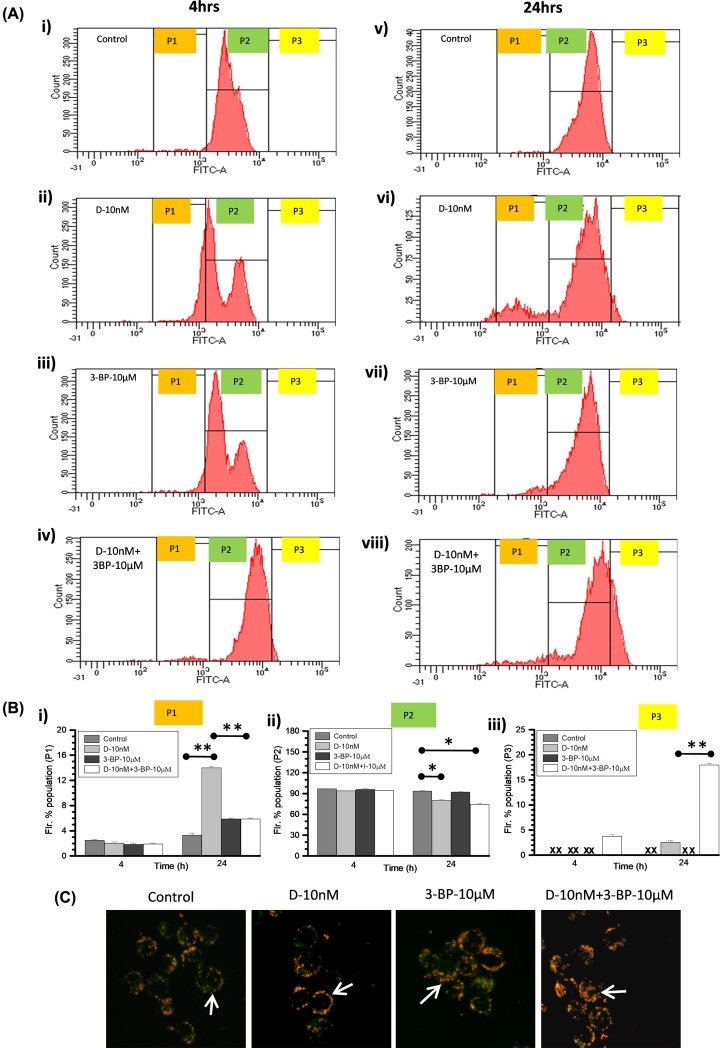
Combined treatment showed hyperpolarized shift of mitochondrial ΔΨm in K-562 cells (**A**) Effect of DNR, 3-BP alone and in combination, on mitochondrial ΔΨm was examined at indicated time points. The histograms present the flow cytometric analysis of DiOC6 staining in K-562 cells. Histogram peak of three gated population presented as P1 (hypo-polarized), P2 (normal as control) and P3 (hyperpolarized) shift of DiOC6 fluorescence. (**B**) Quantitative graph presented as derived information obtained from flow cytometric analysis of P1, P2 and P3 gated population mean fluorescence intensity. Data are presented as mean ± SD (*n*=3) and statistical significance (*P<0.05; **P<0.01) vs. untreated control cells. (**C**) Microscopic evaluation of mitochondrial ΔΨm was carried out using JC-1 at 24 h post treatment. In the photomicrographs, arrows indicate the differential retention of JC-1 as monomers (with green color) and aggregates (with red/orange color) among treatment groups and control. Images were captured under 10× (objective) × 10× (eyepiece) magnification.

A key biochemical checkpoint, the mitochondrial transmembrane potential (ΔΨm) exhibits an important impact on cellular apoptotic event where hyperpolarization leads to cell death [40,41]. Since HK-II present on the outer mitochondrial membrane in the association with VDAC and ANT [33] therefore, we checked if HK-II inhibition by 3-BP altered the ΔΨm in K-562 cells at 4 and 24 h (post treatment). DiOC6 staining carried out followed by flow cytometric analysis where P2-gated cell population of control cells (Figure 7A(i,v)) considered with normal ΔΨm likewise, fluorescence shift in the P-1 and P-3 gated population reflected hypo and hyper ΔΨm, respectively. Nearly similar P2 population observed at initial 4 h among all treatment groups, however at 24 h significant reduction was noted in DNR and CT (Figure 7B(ii)). We observed hypopolarized cell population (P1) at 4 and 24 h in the DNR treated cells (Figure 7A(ii,vi)) as compared with other treatment groups (Figure 7B(i)). However, significantly large hyperpolarized cell population (P-3) observed only in the CT at 4 and 24 h with marginally higher cell numbers in DNR also at 24 h (Figure 7B(iii)). Further in order to strengthen this observation we used the membrane potential-sensitive probe JC-1, which forms JC-1 monomers (with green color) at low membrane potential and J-aggregates (with red/orange color) at higher ΔΨm. At 24 h photomicrograph showing higher retention of J-aggregates in CT suggesting hyperpolarized mitochondrial status (Figure 7C). Together, these observation indicates that the DNR in combination with 3-BP leads to mitochondrial hyperpolarization which enhanced the cell death, i.e. apoptosis in K-562 cells.

## Discussion

The present study revealed that mitochondria-associated HK-II is an important driver of glycolysis-mediated DNR resistance in leukemic cells. The role of up-regulated HK-II has been observed in most cancers and recognized to represent a prominent metabolic characteristic of malignant cells [33]. HK-II localization to outer mitochondrial membrane makes it easier to use ATP generated from mitochondria for glucose phosphorylation. Moreover, by stabilizing MPTP through VDAC binding, it plays an anti-apoptotic effect in malignant cells [23,33]. Since, HK-II exhibits an important role in the both cancer cell metabolism and apoptosis, therefore it was reasonable to speculate that combination of HK-II inhibitor with existing chemotherapeutics may enhance the sensitization in leukemic cells. Earlier it has been demonstrated that besides the presence of HK-I in all the lymphoid cells, cultured leukemic lymphoblast’s characterized by the presence of HK-II [27]. Our data showed an increased glycolysis in K-562 cells as a consequence of increased level and activity of HK-II in these cells. Whereas, the THP-1 cells showed relatively reduced HK-II expression, enzyme activity and glycolysis as compared with K-562 cells. However, no HK-II was detected in normal PBMCs which might be a reason for its normal glycolytic response as compared with both leukemic K-562 and THP-1 cells. These observations validate the direct correlation between HK-II overexpression and an increased glycolytic phenotype of leukemic cells. Simultaneously, we also demonstrated high glycolytic flux of K-562 cells is associated with multifold increased ROS level in these cells. Since, there is a bidirectional link between ROS and glucose metabolism where up-regulation of GLUT proteins stimulate glucose uptake as a part of adaptive mechanism triggered by metabolic or oxidative stress [42]. Excessive, sustained and sublethal ROS level due to compromised oxidative phosphorylation over a prolonged period of time results in activation of HIF-1 (Hypoxia Inducible Factor-1) signaling leading to the over-expression of GLUT-1 transporter protein; thereby inducing a vicious circle activation of ROS-stimulated glucose uptake and *vice versa* [42]. The ROS generation in K-562 cells was four-fold high as compared with THP-1 cells suggestive of K-562 is an aggressive Warburg phenotype [43]. We hypothesized that inhibition of glycolysis and HK-II mitochondrial association using 3-BP, a pyruvate analog and a potent inhibitor of HK-II can weaken the aggressiveness of Warburg phenotype K-562 cells and make it susceptible to anthracycline anticancer agent, DNR [23,44]. We found that 3-BP synergistically sensitized and increased the susceptibility of K-562 cells to 10 nM DNR, by two-fold (29–58% and SI ≥ 1, Figure 2C). Similar observation was noted in acute myeloid leukemic THP-1 cells. Leukemic cells exhibit altered cell metabolism where increased glycolysis linked with drug resistance [11,17]. Interestingly, DNR treatment showed enhanced glucose uptake and mitochondrial complex II activity, which is further verified by nearly three-fold increase in ATP production (Figure 3). This observation is in line with earlier published observation in AML and CML cells, where it was found that Anthracycline drugs (DNR and doxorubicin) treatment results in increased ATP level [45,46]. Warburg phenotype cells are already resistant due to high glycolytic metabolism, however DNR induced hypermetabolic state producing high levels of ATP that may further result in therapeutic resistance by facilitating the repair of DNA and other therapy-induced macromolecular damage [17]. However, CT (DNR + 3-BP) reduced the glycolytic metabolism, mitochondrial complex-II activity and level of ATP as compared with DNR TA. HK-II inhibition mediated reduced ATP level may be one of the prime determining factor behind CT induced enhanced sensitization of K-562 cells.

Inhibition of glycolysis or unavailability of glucose blocks the passage of cell cycle through S-phase [47]. Interestingly, our results also indicate that HK-II inhibition using 3-BP results in substantial decrease in the K-562 cell proliferation through the induction of S-phase (4 h) and G_2_-M phase cell cycle arrest (24 h) augmented by the DNR in the CT (Figure 4). DNR induced cell cycle arrest in G_2_/M phase has been reported in cancer cells including acute myeloid leukemic cells [48]. However, data presented here suggest that the combined DNR and 3-BP treatment leads to profound accumulation of cells in both S phase and G_2_/M phase resulting in significantly higher growth delay. It has been found that mitochondrial dissociation of HK-II is the consequence of 3-BP induced covalent modification followed by the commencement of apoptosis in cancer cells [23,44]. Therefore we examined the correlation between 3-BP induced HK-II dissociation from mitochondria and apoptosis in K-562 cells. The distinctly low level of HK-II in mitochondrial fraction in 3-BP and CT with respect to control and DNR treated sample, suggest that 3-BP inhibits the mitochondrial association of HK-II in K-562 cells. Further reduced level of anti-apoptotic protein BCL-2 and pro-apoptotic protein AIF found in line with enhanced apoptotic population in the CT (Figure 5). These results highlight that DNR induces marginal increase in pro-apoptotic AIF level but no change was observed in the level of anti-apoptotic mitochondrial HK-II or BCL-2, which were found significantly low in 3-BP alone or CT, thereby strengthening the role of glycolytic inhibition and HK-II dissociation from mitochondria in DNR-induced susceptibility in the CT. Subsequently, substantial increase in an intracellular calcium at 4 and 24 h (even after treatment withdrawal) in the CT positively correlated with cytotoxicity observed. The 3-BP induced cell death undergoes mitochondria dependent intrinsic apoptotic pathway coupled with the release of cytochrome *c* which selectively binds to endoplasmic reticulum (ER) inositol (1,4,5) trisphosphate receptor and amplifying calcium-dependent apoptosis [36]. Interestingly, augmentation of apoptotic signal by accumulation of intracellular calcium validates the role of calcium in HK-II inhibition mediated cell death in the CT. The glycolytic inhibition also hampered the response to DNA damage caused by DNR, which was shown as large number of fragmented nuclei and enhanced 53BP1 foci formation in CT at both early (4 h) and late (24 h) time points and evidently correlated with the previous apoptotic events. However, whether this response can be entirely attributed to the suppression of HK-II or other mechanisms of mitochondria to nucleus retrograde signaling involved in the CT remains to be elucidated.

The inhibition of glycolysis and increased intracellular calcium indicated us to investigate the role of mitochondrial membrane potential in the CT induced enhanced apoptosis. Flow cytometric analysis of DiOC6 stained and imaging of JC-I labeled cells revealed that CT resulted in increased ΔΨm, i.e. hyperpolarization (at 24 h post treatment) as compared with control and individual treatment. In the acute leukemic T cells, an increase in the level of mitochondrial cytochrome *c* associated with transient increase in ΔΨm was found to be accompanied by apoptosis [49]. Our observation affirmed that CT induced significant upshift of ΔΨm results in enhanced cell death of leukemic K-562 cells.

**Figure 8:**
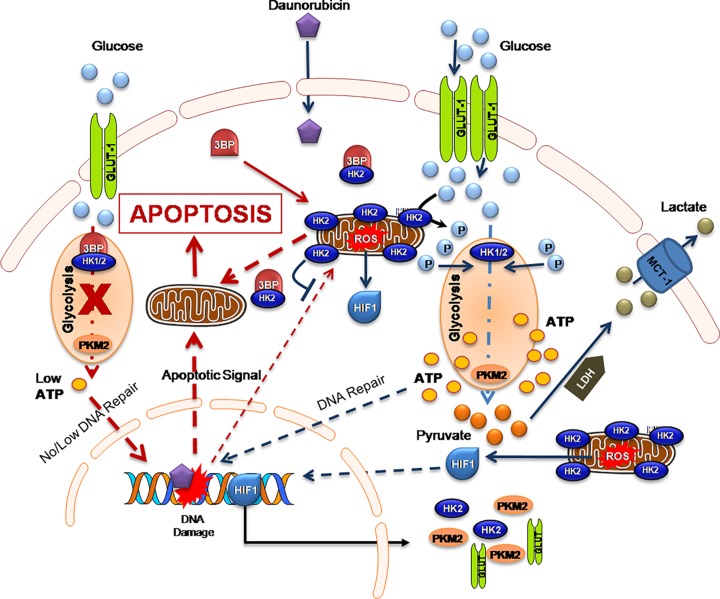
The diagram shows BCR-ABL mutation induced Warburg phenotype in Chronic Myeloid Leukemic cells Mitochondrial ROS mediated inactivation of prolyl hydroxylase (PHD) results in non-hypoxic stabilization and activation of HIF-1 signaling in leukemic cells [49]. HIF-1 mediated GLUT-1, HK-II and PKM-2 over-expression leads to enhanced rate of glycolysis and high ATP level. The high ATP level facilitates the repair of DNA and other therapy induced macromolecular damage. Moreover, enhanced repair also diminishes the magnitude of DNR-mediated DNA damage-induced apoptosis. This intrinsic apoptotic signal is further inhibited by mitochondrial outer membrane associated with HK-II, thereby leading to therapeutic resistance. Inhibition of HK-II by 3-BP reduces the ATP levels by inhibiting glycolysis thereby, hindering DNA repair, which potentiates the intrinsic apoptotic signal in Warburg cell. Further, it also dissociates the HK-II from mitochondria, which allows the release of apoptotic factors from mitochondria and results in induction of intrinsic apoptosis.

In summary, this work provides the evidence supporting HK-II mediated increased glycolytic flux as a key mechanism of DNR induced therapeutic resistance in leukemic cells and therefore, inhibition of glycolysis can sensitize the leukemic cells to therapeutic agents (Figure 8). In fact combined treatment induced mitochondrial HK-II dissociation correlates with substantial attenuation of cell proliferation and enhanced apoptosis marked by retarded DDR and mitochondrial hyperpolarization in leukemic cells (Figure 8). Further the present study suggests that HK-II inhibition in combination with existing therapeutic modalities may provide an actionable therapeutic target in leukemia. Therefore, we propose that this combination should be tried in patients under clinical trial investigation to achieve better therapeutic gain. We also want to emphasize that it is easy to test the drug and metabolic inhibitors susceptibility in leukemic cells from patient *ex vivo*. The treatment induced cell cycle block in S and G_2_/M phases, reduced proliferation, GI and DNA damage markers analysis in isolated cells from patient’s blood sample can elucidate about the susceptibility of leukemic cells to the selected therapeutic agent and combined treatment.

## Abbreviations

AIF: apoptosis inducing factor
ALL: acute lymphocytic leukemia
ATP: adenosine triphosphate
CML: chronic myeloid leukemia
CT: combined treatment
DDR: DNA damage response
DMEM: Dulbecco’s Minimum Essential Medium
DNR: Daunorubicin
EDTA: ethylenediaminetetra acetate
GI: growth inhibition
HK-II: hexokinase-II
H2-DCFDA: dichlorodihydrofluorescein diacetate
LDHA: lactate dehydrogenase A
MDR: multidrug-resistant
MgCl_2_: magnesium chloride
MPTP: mitochondrial permeability transition pore
PBS: phosphate-buffered saline
PI: propidium iodide
ROS: reactive oxygen species
SI: synergistic index
TA: treatment alone
VDAC: voltage-dependent anion channel
2-DG: 2-deoxy-d-glucose
3-BP: 3-bromopyruvate
